# Enhancing Mechanical and Thermal Performance of Injection-Molded PLA via Nucleation and Processing Optimization

**DOI:** 10.3390/polym18131607

**Published:** 2026-06-28

**Authors:** Peng Gao, Max Johnson, Duncan Woodward, Nicholas Gajkowski, Mia Knipe, Anna Armstrong, Leia Kaminsky

**Affiliations:** 1 Materials Science Engineering, Western Washington University, Bellingham, WA 98225, USA; john318@wwu.edu (M.J.); duncan.woodward@hotmail.com (D.W.); nicholas.gajkowski@usm.edu (N.G.); knipem@wwu.edu (M.K.); armstr34@wwu.edu (A.A.); kaminsl2@wwu.edu (L.K.); 2 Polymer Science & Engineering, University of Southern Mississippi, Hattiesburg, MS 39406, USA

**Keywords:** polylactic acid, crystallization, injection molding, nucleating agent

## Abstract

This study examines the effects of 2 wt% orotic acid (OA) nucleation and injection molding conditions on the crystallization behavior and thermo-mechanical performance of polylactic acid (PLA). Differential scanning calorimetry and X-ray diffraction revealed that 2 wt.% OA accelerates crystallization, enabling molded PLA to achieve crystallinity levels as high as 52–53% under low packing pressure and long hold time. Mechanical testing showed that tensile modulus increased with longer hold time, while tensile strength decreased due to constrained relaxation in the skin layer. Flexural strength increased with packing pressure, whereas flexural modulus decreased as the degree of crystallinity decreased under higher pressure conditions. Heat deflection temperature (HDT) showed the greatest sensitivity to processing, rising from 58 °C to 100–131 °C in optimized PLA–OA samples. The highest HDT values occurred under conditions that promoted both high crystallinity and extended lamellar development with strong α-phase formation. These results demonstrate that combining OA nucleation with controlled injection molding enables high-crystallinity, high-HDT PLA without post-annealing, offering a viable route for producing thermally stable PLA components suitable for hot-fill and reheatable food packaging applications.

## 1. Introduction

The global demand for sustainable and environmentally friendly materials has generated great interest in bio-based polymers as alternatives to fossil-fuel-derived plastics. Among these, polylactic acid (PLA) has gained particular interests due to its compostabilty, bio-origin from renewable feedstocks such as corn and sugarcane, and compatibility with standard polymer processing techniques, such as injection molding, extrusion, compression molding, etc. [1]. As regulations, laws, and public pressure continue to drive a transition toward circular materials with low-carbon footprint and product life cycles, PLA stands out as a key competitor in sustainable manufacturing strategies across sectors ranging from packaging to biomedical devices [2].

However, despite its environmental benefits and commercial availability, PLA’s mechanical limitations and poor thermal stability restrict its application in engineering environments. In the packaging industry and other commodity plastic industries, fossil-fuel-derived polymers such as polypropylene (PP) and polyethylene (PE) are widely used in food containers, automotive interiors, and durable consumer goods due to their excellent toughness, ductility, and heat resistance, with HDTs typically ranging from 100 °C to 130 °C [3]. Polyethylene terephthalate (PET), a common material for beverage bottles and thermoformed packaging, provides products with high clarity, strength, and dimensional stability, and has a glass transition temperature (Tg) near 80 °C and HDTs often above 110 °C [4].

In contrast, PLA typically softens and deforms near its Tg (~60–65 °C), with HDTs below 60 °C for its most applications, unless additional post-fabrication treatments are introduced [5,6,7]. Furthermore, while PP and PE can sustain repeated mechanical loading due to their toughness and strain-hardening behavior, PLA is inherently brittle, with low impact strength and poor elongation at break under both ambient and elevated temperature conditions. These disadvantages limit PLA’s use in containers that hold hot material (e.g., single-use hot coffee cups), microwavable food trays, reusable and hot-washable utensils, and semi-structural components. To close this performance gap, it is essential to enhance PLA’s thermal and mechanical performance. One of the methods is increasing its crystallinity during or immediately after manufacturing.

As a semicrystalline polymer, PLA’s mechanical and thermal behavior is strongly influenced by its crystalline content and morphology. A higher degree of crystallinity correlates with improved stiffness (Young’s modulus), tensile strength, dimensional stability, and thermal resistance, which are critical for high-performance applications [7,8,9]. However, PLA exhibits a slow crystallization rate. Under standard injection molding conditions, PLA cools too rapidly to develop a substantial degree of crystallinity, resulting in largely amorphous molded parts. Amorphous PLA usually softens near its glass transition temperature (Tg~60–65 °C) and suffers from deformation under modest thermal or mechanical loads. Reported tensile strengths for PLA vary from as low as 21 MPa to as high as 150 MPa, with Young’s modulus ranging from 0.35 to 4.14 GPa, depending on whether the material is amorphous or semicrystalline, and whether it is composed of poly(l-lactic acid) (PLLA), poly(dl-lactic acid), or a racemic mixture [5,9,10,11]. As summarized by Van de Velde et al., semicrystalline L-PLA typically achieves much higher modulus and strength values than amorphous DL-PLA, which lacks a defined melting point and crystalline domains [12]. Crystalline regions contribute to increased stiffness, higher strength, and better thermal resistance, while amorphous PLA tends to be softer, less brittle, and more thermally sensitive. This large property variation highlights the importance of controlling crystallization during processing if consistent mechanical performance is desired. To address this, significant research has been devoted to identifying processing techniques and formulation strategies that increase the degree of crystallinity in PLA, either during molding or through secondary treatment. Several processing pathways have been developed to promote crystallization in PLA, both in situ during molding and through post-processing approaches. These strategies generally fall into three categories: post-mold annealing, incorporation of nucleating agents, and shear-induced crystallization during processing.

Annealing is a post-processing thermal treatment that enhances the crystallinity of PLA by heating the polymer above its glass transition temperature (Tg) but below its melting point, typically around 100–120 °C. The exposure to higher temperatures and the introduction of additional energy after fabrication increase the mobility of polymer chains in the amorphous phase, allowing them to reorganize into more ordered crystalline structures. For neat PLA, annealing primarily promotes the growth of existing crystalline nuclei, increasing crystallinity and improving mechanical properties such as stiffness and strength [5,6,13,14,15,16].

The effectiveness of annealing depends on both temperature and duration, with higher temperatures generally accelerating crystallization. For example, Gao et al. reported that neat PLA’s crystallization half-time decreased from 34.2 min at 80 °C to 2.14 min at 110 °C, while the degree of crystallinity increased from 2.5% to 50.5% [17]. Studies also show that crystallinity above 40% can be achieved at 80 °C within 30 min, while at lower temperatures (e.g., 65 °C), it requires hours [9]. These results highlight annealing’s potential to transform initially amorphous molded PLA parts into semicrystalline structures with significantly enhanced thermal and mechanical performance.

Annealing is a practical and effective method for improving the in-use properties of neat PLA, particularly in applications requiring dimensional stability and elevated service temperatures. However, its reliance on a secondary processing step may limit throughput in high-volume manufacturing environments and may also affect the dimensional stability of the product due to shrinkage.

Nucleating agents are widely used to accelerate crystallization in PLA by providing sites for heterogeneous nucleation, thus reducing the crystallization half-time and increasing the overall degree of crystallinity. In neat PLA, the addition of nucleating agents can improve processing efficiency and enhance thermal and mechanical properties. These additives work by increasing the density of crystalline nuclei, which leads to smaller and uniformly distributed crystallites during cooling or annealing.

Among the most studied nucleating agents for neat PLA are inorganic fillers such as talc, calcium carbonate, and nano-clays [8,13,18,19,20]. Talc, in particular, has demonstrated high nucleation efficiency, often increasing crystallinity by more than 30% under suitable conditions [19]. Carbon-based nanomaterials, such as carbon nanotubes (CNTs) and graphene oxide, have also been explored [21,22]. These nanofillers not only act as nucleation sites but can also improve mechanical reinforcement due to their high aspect ratio and stiffness. In addition, bio-based nucleating agents like orotic acid (OA) offer a sustainable alternative [23,24]. For example, Gao et al. showed that just 1 wt% of OA in neat PLA reduced the crystallization half-time from 2.1 min to 1.5 min at 110 °C and increased crystallinity from 6.5% to over 14.7% under quiescent conditions [17]. Unlike many inorganic fillers, OA does not compromise the compostability of PLA and remains compatible with bio-based applications. The use of nucleating agents is a well-deployed strategy to increase the crystallinity of neat PLA in the industry.

Shear-induced crystallization occurs when shear stress is applied to a polymer melt during processing, causing the polymer chains to align and crystallize more readily. Several studies have confirmed that PLA will benefit from shear-enhanced crystallization. Jalali et al. reported that applying shear at moderate rates (10–33 s^−1^) reduced crystallization induction time and favored the formation of stable α-phase crystals [25]. Bojda et al. observed that increasing shear from 10 to 50 s^−1^ intensified crystallization, although too-high shear (e.g., 100 s^−1^) significantly reduced its effectiveness [26]. These findings suggest that an optimal and controlled shear window would promote a desirable morphology in neat PLA and increase the degree of crystallinity [27,28]. Shear-controlled orientation molding (SCORIM) also demonstrated effectiveness; Altpeter et al. showed that applying oscillatory shear during the holding phase increased crystallinity from 4% to 21% in injection-molded PLA [29]. Recent innovations such as vibration-assisted injection molding (VAIM) further demonstrate the potential of shear to improve PLA performance [30]. In VAIM, mold wall vibrations enhance shear at the melt–mold interface, enabling highly crystalline structures at lower mold temperatures and reduced cycle times. While most studies apply VAIM to nucleated PLA systems, the principles could be extended to neat PLA, offering a promising route to improve thermal stability, stiffness, and process efficiency without secondary treatments.

Most prior research tends to analyze these enhancement strategies in isolation. However, in real-world manufacturing, the interplay between nucleating agents, shear conditions, and thermal conditions during manufacturing must be analyzed as a coupled system. Injection molding is the most widely used fabrication technique for PLA products. It presents a unique opportunity to induce crystallization in situ by optimizing and controlling injection velocity, packing pressure to adjust shear, mold temperature, and hold time to adjust thermal exposure.

This study builds upon previous work by investigating the combined effects of a bio-based nucleating agent (2 wt% orotic acid) and injection molding parameters on the crystallization behavior, mechanical performance, and thermal stability of PLA. A full design of experiments (DOE) approach was used to vary shear conditions, packing pressure, and holding time across two material systems: neat PLA and PLA with 2 wt% orotic acid. The primary focus of this paper is to quantify how crystalline development affects the mechanical and thermal performance of PLA by controlling the processing conditions and the adoption of the nucleating agent.

## 2. Materials and Methods

### 2.1. Material Selection

The material of interest for this research is PLA Ingeo 3100HP synthesized by NatureWorks LLC (Plymouth, MN, USA). PLA 3100HP is a semicrystalline injection molding grade PLA material widely used in packaging and utensil industries. The material is a low-to-mid-viscosity injection molding material. The key physical and thermal properties are presented in Table 1.

Orotic acid (OA) from MilliporeSigma (Burlington, MA, USA) was selected as the nucleating agent used to promote crystallization. It was received in the form of a white powder and was stored in a humidity-controlled environment protected from UV radiation.

### 2.2. Compounding Procedure

PLA pellets were dried at 50 °C for 6 h under vacuum, and the orotic acid was dried at 100 °C for 4 h before compounding. A co-rotating twin-screw extruder (Mio27/6l-400, L/D ratio 40:1, Leistritz, Nürnberg, Germany) was used to compound the PLA with 2 wt% orotic acid. The barrel temperature profile was set from 160 °C (feed zone) to 190 °C (die), with a screw speed of 150 rpm and a feed rate of approximately 5 kg/h. The extrudate was water-cooled (20–25 °C) and pelletized. All compounded materials were re-dried at 40 °C for over 12 h before molding. The compounded batch was named PLA-2OA, and neat PLA 3100HP was also molded for comparison, and the batch was named neat PLA.

The crystallization behavior and morphology of the same PLA-2OA formulation under quiescent conditions have been previously characterized using DSC, XRD, and SEM and are reported in previous research [17]. The present study focuses on the effects of injection molding processing parameters on the crystallization development and thermo-mechanical performance of the compounded material.

### 2.3. Injection Molding of Samples and Design of Experiments (Doe)

All material characterization was performed on tensile specimens molded using a double-cavity tool installed on a Sumitomo SE75EV-C160 injection molding machine (Chiba, Japan). Three processing parameters were varied: injection velocity (V), pack/hold pressure (P), and holding time (T). Each parameter was tested at two levels, one (+) and one (−), to a selected midpoint. To assess the influence of crystallinity and thermal history, mold temperatures were set to 27 °C for neat PLA and 95 °C for PLA-2OA.

A factorial design of experiments (DOE) was used to generate eight processing combinations for each material system, as listed in Table 2. Each condition was run for 18 molding cycles, resulting in 36 tensile bars per combination for subsequent characterization. Each DOE run was repeated 8 times to produce replicates for different characterization methods. The full experimental plan is presented in Table 3. The production order was randomized to reduce variability.

### 2.4. Characterization Techniques

A set of characterization techniques was employed to evaluate both the neat PLA and PLA-2OA. These included thermal, mechanical, structural, and morphological analyses. Each method requires specific sample preparation protocols to ensure data accuracy and consistency.

#### 2.4.1. Thermogravimetric Analysis (TGA)

Thermal stability and thermal degradation behavior were assessed using a TA Instruments Discovery 5500 (New Castle, DE, USA) thermogravimetric analyzer. Small segments (~10 mg) were cut from the gauge section of tensile specimens and subjected to a dynamic heating cycle in which the temperature was increased from 25 °C at a rate of 50 °C per minute up to 600 °C.

#### 2.4.2. Differential Scanning Calorimetry (DSC)

DSC measurements were carried out on a TA Instruments Discovery X3 (New Castle, DE, USA) calorimeter. Three samples, each weighing between 5.0 and 8.0 mg, were prepared from a single tensile bar using plastic cutters. A standard heat–cool–heat cycle from 20 °C to 240 °C was performed at a rate of 10 °C per minute. Crystallinity was calculated from the first heating cycle using Trios software (version 5.9.1.32), following the equation:

(1) $$crx\%=\frac{∆H_{m}-∆H_{cc}}{{∆H}_{100\%crxn}}\times 100\%$$

where ∆H_m_ is the melting enthalpy and ∆H_cc_ is the cold crystallization enthalpy of the tested sample. ∆H_100%crxn_ is the melting enthalpy of a PLA crystal of infinite size. The latter was assumed to be 93 J/g, as obtained from the literature.

#### 2.4.3. X-Ray Diffraction (XRD)

The crystalline structure of the samples was analyzed using a Rigaku MiniFlex 6G (The Woodlands, TX, USA) diffractometer. Scans were conducted from 10° to 40° (2θ) at 0.02° intervals, with a dwell time of 120 s per step. The resulting diffraction patterns were evaluated to identify and distinguish PLA morphologies.

#### 2.4.4. Tensile Testing

Tensile properties were measured using a Shimadzu Autograph AGS-X Series (Kenmore, WA, USA) universal testing machine with pneumatic grips pressurized to 60 psi. The test span was set at 100 mm, and the crosshead moved at a constant speed of 5 mm/min. Testing adhered to ASTM D638 [31], and five specimens per processing condition were tested to capture batch variability.

#### 2.4.5. Flexural Testing

Flexural strength was assessed using the same Shimadzu load frame (Kenmore, WA, USA), configured for three-point bending. The test setup featured a support span of 56 mm and a loading rate of 10 mm/min. Five tensile bars from each batch were used for this evaluation.

#### 2.4.6. Dynamic Mechanical Analysis (DMA)

Dynamic mechanical behavior was examined in a three-point bending configuration using a TA Instruments Q800 DMA (New Castle, DE, USA). Samples were heated from room temperature to 160 °C at a heating rate of 2 °C/min under a constant applied load. The resulting flexural stress was approximately 0.45 MPa, depending on specimen dimensions. Specimen displacement was continuously recorded throughout the test. For samples that maintained dimensional stability throughout the temperature ramp, the final deflection at 160 °C was reported. For samples exhibiting excessive thermal softening, the displacement exceeded the DMA auto-stop limit (approximately 10 mm) before reaching 160 °C, and the corresponding temperature was recorded as the heat deflection temperature (HDT).

## 3. Results and Discussion

### 3.1. Neat PLA

#### 3.1.1. Thermal Stability and Thermal Degradation

**Figure 1:**
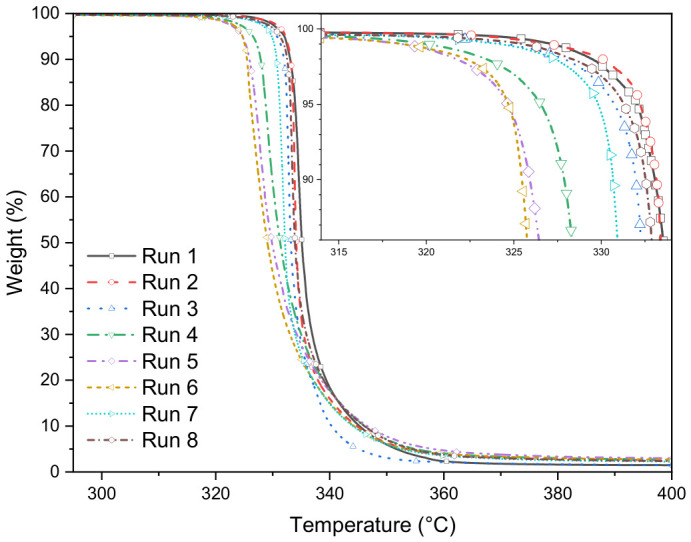
Thermal degradation behavior of neat PLA samples.

The degradation behavior of the neat PLA sample is presented in Figure 1, and the degradation onset temperatures are summarized in Table 4. The degradation onset temperatures (T_onset_) of all samples are distributed in the range of 324.86–333.33 °C, while the 90% weight loss temperatures (T_90_) values range from 340.21 to 351.49 °C. The differences between T_onset_ and T_90_ are in the range of 15–25 °C, indicating a rapid single-stage degradation behavior leading to minimal char formation.

The statistical analysis suggested that none of the processing parameters were significant for the degradation onset temperature. Injection velocity, packing pressure, and pack-and-hold duration, within the studied range, did not induce significant molecular weight reduction through chain scission. This suggests that the thermal–mechanical history during molding was insufficient to cause permanent structural degradation for the neat PLA samples.

#### 3.1.2. Degree of Crystallinity

A summary of the melting enthalpy, melting point, cold crystallization temperature and enthalpy is presented in Table 5, and the degree of crystallinity is presented in Table 6.

**Figure 2:**
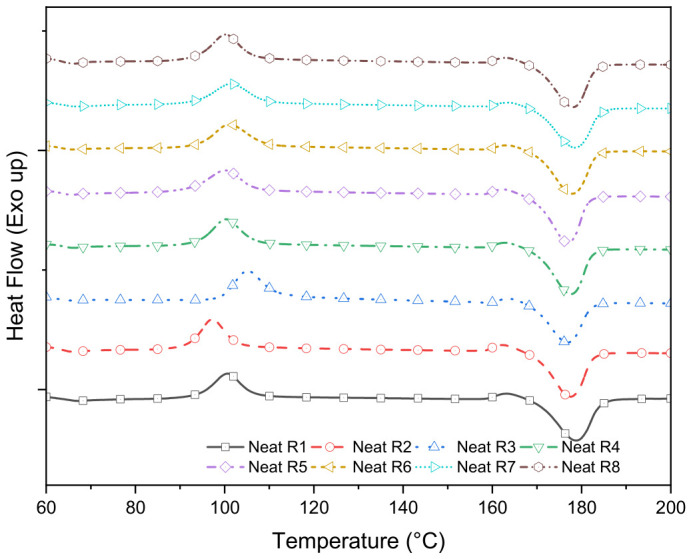
Crystallization behavior of neat PLA samples.

Differential scanning calorimetry of molded specimens indicates that the parts were not fully crystallized as expected (c.f.: Figure 2). Cold crystallization enthalpies (ΔH_cc_) were 21.6–23.7 J/g, and cold crystallization temperatures (T_cc_) ranged from ~100–102 °C (one sample at 96.8 °C), demonstrating that a significant amorphous fraction remained after molding and crystallized upon reheating. Melting enthalpies (ΔH_m_ = 39.6–43.9 J/g) and melting temperatures (T_m_ ≈ 175–179 °C) were narrowly distributed, indicating that the crystalline morphology and the final crystalline perfection produced during the test were consistent across all DOE runs. The relatively small run-to-run variation in ΔH_m_ and T_m_ suggests the final crystalline phase is insensitive to the tested injection-parameter window, while modest variability in ΔH_cc_ and T_cc_ points to slight differences in nucleation density or cooling-rate microenvironments during molding.

An ANOVA study for the degree of crystallinity suggests that all three DOE factors were not significant for the degree of crystallinity for neat PLA. *p*-values for injection velocity, packing pressure, and holding duration were 0.73, 0.63, and 0.35, respectively.

The average degree of crystallinity of all DOE runs varied between approximately 17% and 23%, with standard deviations reflecting moderate consistency. These findings indicate that within the studied processing window, the molding parameters had minimal influence on the overall crystalline fraction developed during solidification. This supports the interpretation that the thermal history and cooling rates dominant in the molding process are relatively stable and not strongly affected by changes in injection velocity, packing pressure, or hold time within this range.

#### 3.1.3. Crystalline Morphology

**Figure 3:**
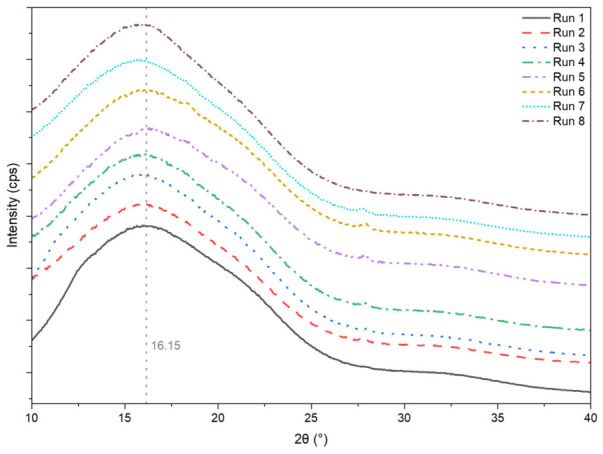
X-ray diffraction (XRD) pattern for neat PLA samples.

X-ray diffraction (XRD) analysis was conducted on specimens from all DOE conditions. The neat PLA samples exhibited a predominantly amorphous structure with limited crystalline development. As shown in Figure 3, a broad diffraction feature centered near 2θ ≈ 16.15° was observed in the XRD patterns. The low intensity and broad nature of this peak indicate limited crystalline development rather than a fully amorphous morphology. This observation is consistent with the DSC results, which measured crystallinity values ranging from approximately 17% to 22%. The broad diffraction feature suggests that the crystalline domains are limited in quantity and/or structural perfection, resulting in weak diffraction intensity despite measurable crystallinity. This observation is consistent with the low to moderate degree of crystallinity measured by DSC for all neat specimens in this study. Similar results have been reported in the literature for PLA processed under rapid and low temperature (<40 °C) cooling conditions, where insufficient time for chain organization during solidification results in amorphous morphologies [32,33,34]. The agreement between the DSC and XRD data supported the conclusion that the processing parameters used here yielded low-crystallinity molded parts. The injection velocity, packing pressure, and holding duration did not have a significant contribution to crystallization development for neat PLA materials.

#### 3.1.4. Tensile Properties

**Figure 4:**
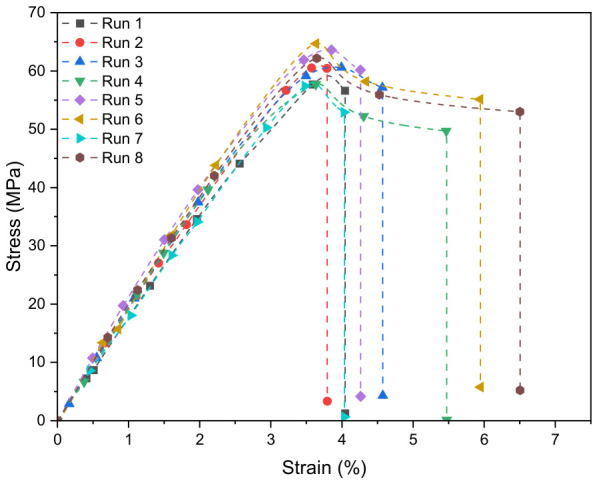
Stress vs. strain curve of neat PLA samples.

The tensile results of all DOE runs are presented in Table 7, and the average tensile curves of the neat PLA specimens are presented in Figure 4.

The tensile behavior of neat PLA was evaluated under varying injection molding parameters, with ultimate tensile stress (UTS) ranging from 56.43 MPa to 67.57 MPa and Young’s modulus from 1.85 GPa to 2.54 GPa. Maximum elongation varied minimally (3.61–3.93%), indicating relatively stable ductility across processing conditions. Standard deviations for UTS (0.94–3.05 MPa) and Young’s modulus (0.04–0.18 GPa) confirm the reproducibility of the measurements, with slightly higher variability observed in the highest UTS runs (Run 5), possibly due to minor differences in cavity filling or local molecular orientation.

Statistical analysis results quantitatively support these trends. For UTS, injection velocity (*p*-value = 0.026), pack pressure (*p*-value = 0), and hold time (*p*-value = 0.031) were all significant at the 95% confidence level, indicating that all three factors meaningfully influence tensile strength. Higher injection velocity and higher pack pressure generally increased UTS, while longer hold times tended to slightly reduce tensile strength. Young’s modulus was significantly affected by injection velocity (*p*-value = 0.044) but not by pack pressure (*p*-value = 0.103) or hold time (*p*-value = 0.414), suggesting that stiffness is primarily controlled by velocity-induced molecular alignment.

**Figure 5:**
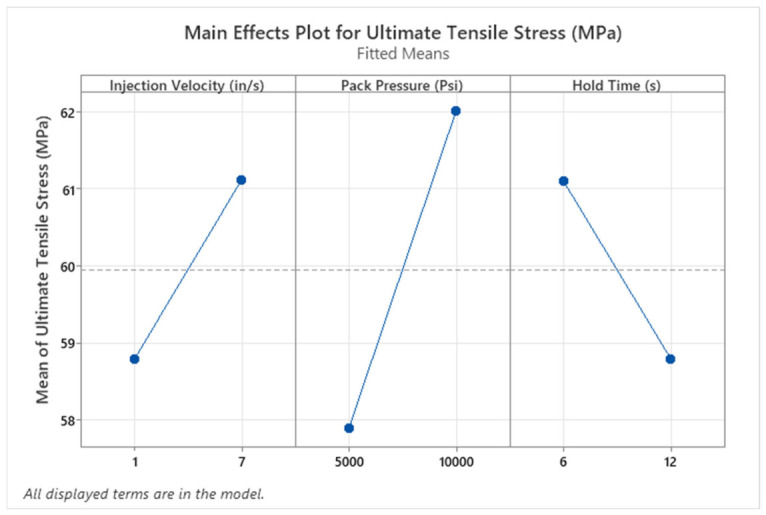
Main effects factorial plots for ultimate tensile stress of neat PLA.

**Figure 6:**
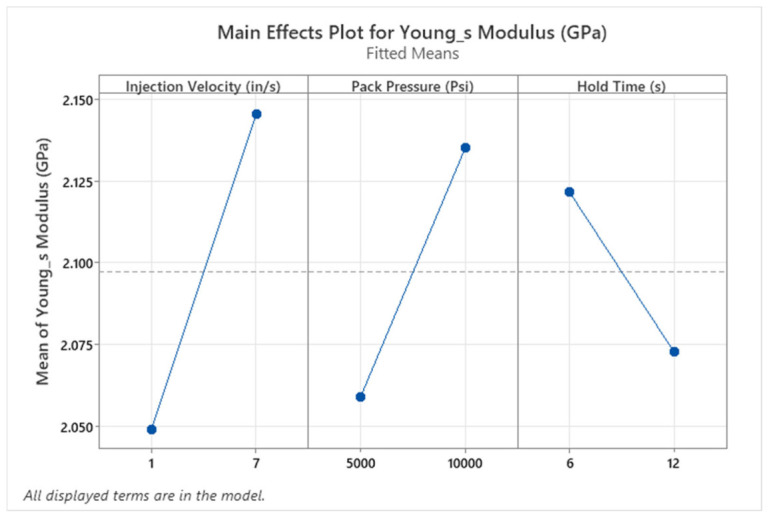
Main effects factorial plots for Young’s modulus of neat PLA.

As shown in Figure 5 and Figure 6, high injection velocities promote polymer chain alignment along with the flow direction, enhancing load-bearing capacity and tensile strength. High pack pressures reduce void formation and improve inter-chain stress transfer, further increasing UTS. Longer hold times may allow partial relaxation of these oriented chains, reducing the benefit of velocity and pressure on strength, which aligns with the slight decrease in UTS observed in extended hold runs. In semicrystalline polymer materials, higher relaxation would result in a higher degree of crystallinity. However, the crystallization rate of neat PLA is slow; thus, 6 s or 12 s of holding time did not enhance the crystallization behavior to improve the UTS. The observation is confirmed by the low degree of crystallinity (<25%) across all DOE runs. Young’s modulus trends were similar to UTS for velocity, supporting the hypothesis that molecular alignment also stiffens the material, while the limited influence of hold time on modulus suggests that orientation effects dominate over densification in controlling stiffness. The low degree of crystallinity confirmed by DSC and XRD indicates that these mechanical improvements arise primarily from processing-induced orientation and packing rather than from crystalline reinforcement [35,36].

#### 3.1.5. Flexural Properties

**Figure 7:**
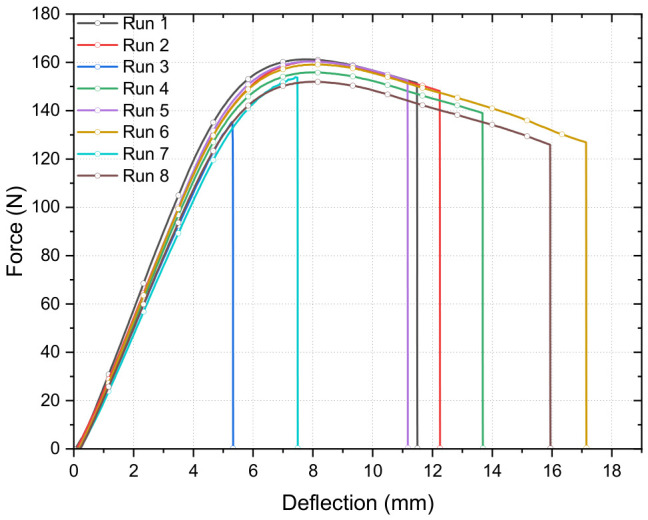
Deflection vs. force curve of neat PLA samples.

The flexural properties of the neat specimens are presented in Table 8 and Figure 7.

The flexural test data demonstrated consistent mechanical performance across replicate samples, with relatively low standard deviations (<5% relative standard deviation for modulus and <4% for strength), suggesting good repeatability of the injection molding process. Flexural modulus values ranged from 3.09 to 3.49 GPa, while flexural strength spanned a wider range of 86.5–116.7 MPa depending on processing conditions. Notably, specimens molded under high injection velocity and high pack pressure exhibited the highest strength values, exceeding 115 MPa, while low pack pressure at high velocity produced strengths below 95 MPa. This spread indicates that flexural strength was more sensitive to processing variations than flexural modulus.

**Figure 8:**
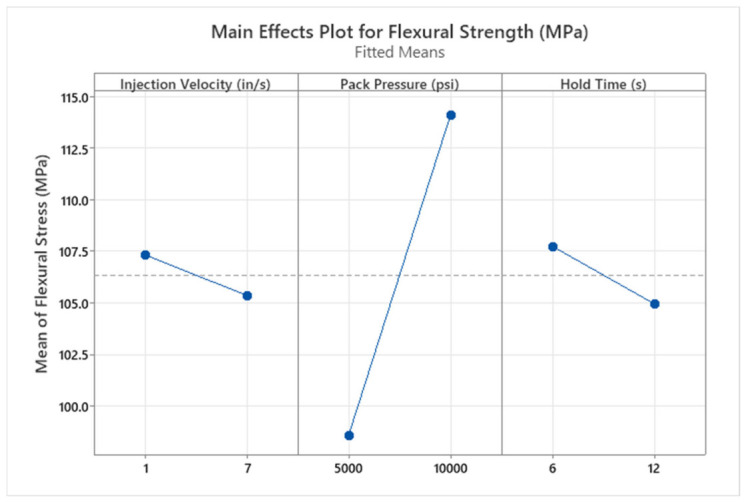
Main effects factorial plots for flexural strength of neat PLA.

**Figure 9:**
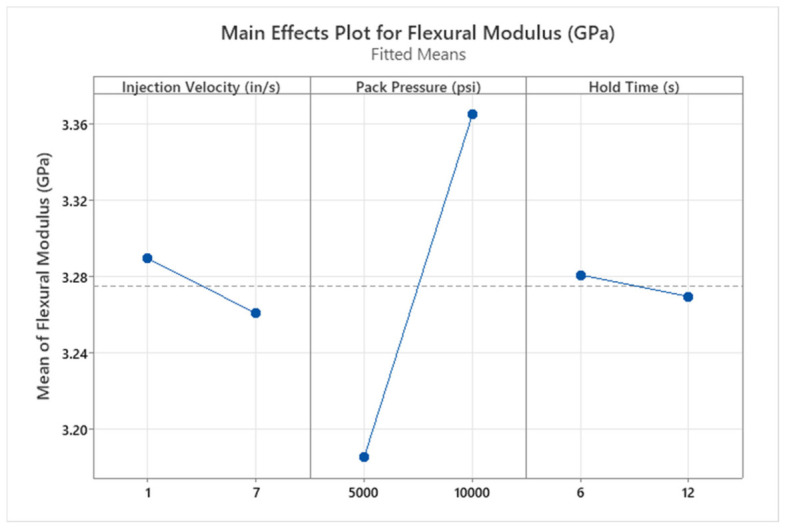
Main effects factorial plots for flexural modulus of neat PLA.

ANOVA results confirmed these observations. For flexural modulus, pack pressure (*p* < 0.001) was significant, while injection velocity (*p* = 0.265) and hold time (*p* = 0.657) were not. The increase in modulus at higher pressure can be attributed to better packing density, which reduces voids and enhances stiffness. As shown in Figure 8 and Figure 9, all three processing parameters were significant. Strength was maximized only when high injection velocity was paired with high packing pressure, indicating that both filling and packing stages contribute to forming a dense and cohesive surface layer in neat PLA. High velocity improves melt flow and promotes more uniform cavity filling, but without sufficient packing pressure, residual voids or incomplete consolidation can limit strength. When high pressure is applied immediately after high-speed filling, the melt is compressed more effectively into the skin and subsurface regions, reducing defects and increasing density where flexural stresses are concentrated [37,38].

#### 3.1.6. Heat Deflection Temperature

The heat deflection temperature (HDT) results for neat PLA are summarized in Table 9. Across all DOE conditions, HDT values ranged between 57.2 and 58.8 °C, remaining well below 60 °C. These values fall within the glass transition temperature (Tg) range of PLA (55–65 °C), consistent with the differential scanning calorimetry (DSC) data that confirmed a predominantly amorphous morphology. The narrow spread of HDT across processing conditions suggests that injection velocity, packing pressure, and hold time had minimal influence on thermal resistance under the tested range, further supporting the hypothesis that crystallinity, rather than flow history, is the governing factor for heat deflection temperature and dominates the service temperature of the molded product.

To further illustrate the relationship between crystallinity and thermal performance, the crystallinity values obtained from DSC are presented alongside the HDT results in Table 9. Across all DOE conditions, the neat PLA samples exhibited consistently low crystallinity levels, corresponding to HDT values near the glass transition temperature. The absence of substantial variation in either crystallinity or HDT further supports the conclusion that the processing conditions investigated were insufficient to induce meaningful crystal development in neat PLA during molding.

The low HDT values indicate that the molded samples were unable to sustain dimensional stability once heated into the glass transition region. Upon reaching Tg, chain mobility increased substantially, leading to rapid softening and deformation. The test specimens consistently reached > 4 mm displacement before bottoming out on the HDT fixture, a behavior typical of amorphous PLA where no semicrystalline lamellae are present to resist chain slippage. This observation is in line with Pan et al. [39], who reported that amorphous PLA lacks sufficient crystalline reinforcement to provide thermal load-bearing capability above Tg.

The mechanistic link between thermal and mechanical data is evident: the same amorphous morphology that limited tensile ductility and flexural resilience also constrained thermal resistance. In semicrystalline systems, crystalline lamellae act as rigid nodes that maintain structural integrity above Tg, thereby elevating HDT [40]. Without such crystalline reinforcement, the PLA studied here exhibited rapid thermal softening and deformation, consistent with the literature on injection-molded amorphous PLA [41].

Several studies support this morphology-driven explanation. Tábi et al. demonstrated that annealed PLA samples, which exhibited crystallinities above 25%, showed a significant increase in HDT (from ~55 °C to over 110 °C) [42]. Similarly, Di Lorenzo and Androsch emphasized that crystallization kinetics strongly govern the thermal-mechanical coupling of PLA, where higher crystallinity translates to simultaneous improvements in modulus, toughness, and HDT. In the present study, the consistently low HDT values closely correspond to the low crystallinity levels measured by DSC across all DOE conditions. Despite variations in injection velocity, packing pressure, and hold time, neither crystallinity nor HDT changed substantially, indicating that processing alone was insufficient to promote crystal development in neat PLA. These findings reinforce the conclusion that crystallinity, rather than the investigated processing parameters, is the primary factor governing thermal resistance and service temperature in the molded PLA components.

### 3.2. PLA-2OA

#### 3.2.1. Thermal Stability and Thermal Degradation

**Figure 10:**
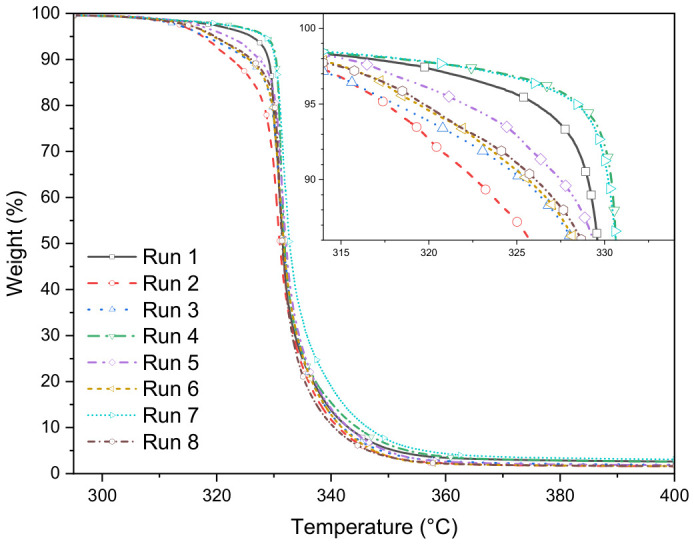
Thermal degradation behavior of PLA-2OA samples.

Thermogravimetric analysis (TGA) of PLA-2OA showed consistent thermal degradation behavior across all DOE runs. As shown in Table 10 and Figure 10, the onset degradation temperature (T_onset_) ranged from 327.9 to 330.4 °C, with an average of ~329 °C, while the temperature corresponding to 90% mass loss (T_90_) spanned 340.6 to 346.4 °C. The narrow T_onset_ range suggests that the addition of 2OA did not alter the degradation mechanism of PLA, which is dominated by random chain scission of ester linkages, but it did provide slightly greater uniformity in thermal stability compared to neat PLA.

The relatively small gap of ~12–17 °C between T_onset_ and T_90_ indicates a rapid degradation process once decomposition is initiated, consistent with the one-step weight-loss profile typical of PLA [1]. Compared to neat PLA, PLA-2OA exhibited a similar onset but a marginally more consistent high-temperature degradation profile, as reflected in the tighter clustering of T90 values. This suggests that 2OA incorporation neither delays nor accelerates the main decomposition pathway but may reduce heterogeneity in chain scission events across processing conditions.

As observed in Section 3.1.1 for neat PLA, injection velocity, packing pressure, and hold time did not produce statistically meaningful effects on T_onset_ or T_90_. This reinforces the conclusion that thermal degradation is intrinsic to the PLA backbone and only minimally influenced by typical injection molding process variations. Similar observations have been reported in the literature for neat PLA and PLA blends, where onset temperatures generally fall between 325 and 335 °C and the degradation proceeds through a rapid, single-step process [43,44,45].

#### 3.2.2. Degree of Crystallinity

**Figure 11:**
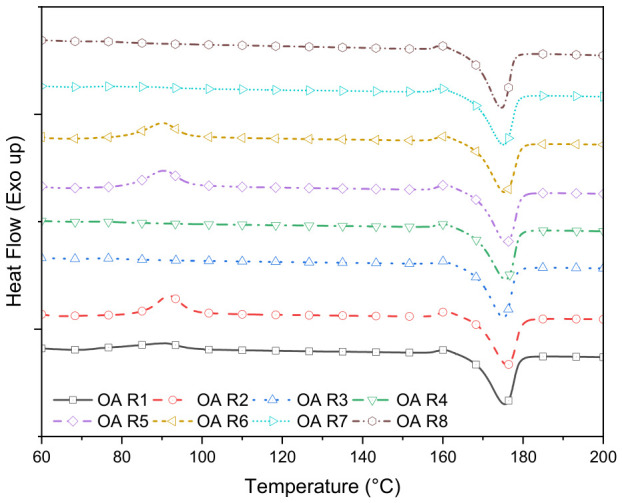
Crystallization behaviors of PLA-2OA samples.

The crystallization behaviors of PLA-2OA samples are summarized in Table 11 and Figure 11, and the degree of crystallinity of PLA-2OA samples is calculated in Table 12.

The calculated degrees of crystallinity for PLA-2OA display a wider distribution across DOE runs, with four specimens clustering in the mid-20% range (≈23–29%) and four specimens exhibiting substantially higher crystallinity (≈52–53%). The overall mean crystallinity is 39.1% with a standard deviation of 14.0%, which is higher and significantly more variable than the neat-PLA values (≈20.0% with a standard deviation of 2.9%) reported in Section 3.1. This indicates that the 2OA additive substantially increases PLA’s propensity to crystallize under certain processing conditions, while the final crystallinity remains highly sensitive to molding history.

**Figure 12:**
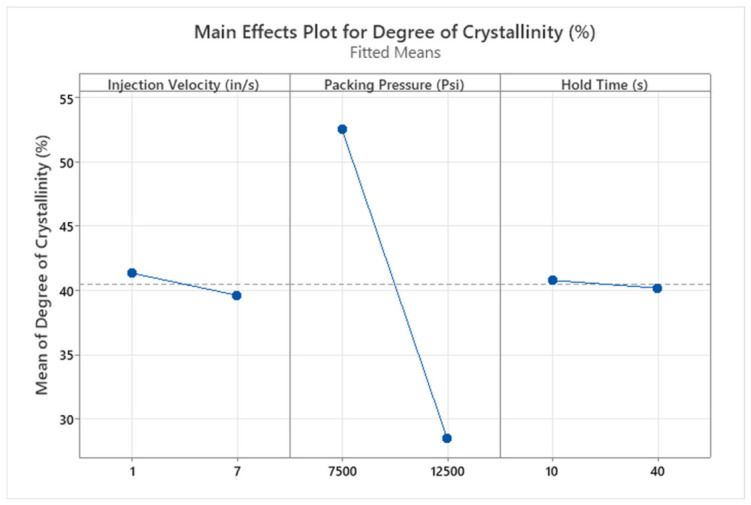
Main effects factorial plots for degree of crystallinity of PLA-2OA samples.

The full factorial DOE analysis suggests that the pack pressure is statistically significant for the degree of crystallinity in PLA-2OA samples. The *p*-value of pack pressure is 0.002; meanwhile, the *p*-values of injection velocity and hold time are 0.635 and 0.866, respectively. As shown in Figure 12, the low packing pressure of 7500 psi resulted in highly crystallized samples with an average degree of crystallinity of 52.2%, while high packing pressure led to a degree of crystallinity of 28.8%.

The crystallization behavior observed in PLA-2OA during injection molding is inherently complex due to the simultaneous presence of high shear rates, steep temperature gradients, and rapid pressure fluctuations [46,47,48]. Lower packing pressure tends to extend the effective cooling window and maintain greater chain mobility during the crystallization interval, promoting lamellar development and yielding a higher degree of crystallinity. Conversely, high packing pressure enhances interfacial heat transfer and accelerates solidification by packing molecules tightly onto the colder mold (95 °C, which is lower than the crystallization temperature of PLA at 103–110 °C), leading to vitrification before extensive crystal growth can occur [49,50]. This interplay between pressure and cooling rate has been reported across multiple semicrystalline systems. These findings support the hypothesis that, in PLA-2OA, packing pressure primarily affects the crystal growth stage rather than nucleation itself, with higher packing pressure resulting in lower overall crystallinity.

Compressing molecules at high packing pressures can also reduce free volume, constrain molecular motion and limit lamellar thickening and growth. Hieber and Shen (1980) developed a pressure-dependent crystallization model for isotactic polypropylene (iPP), demonstrating that while higher pressure enhances nucleation, it simultaneously reduces chain mobility, thereby slowing crystal growth [51]. The interaction between shear-induced nucleation and pressure-limited growth further complicates this behavior. Tang et al. examined PLA and PLA-carbon nanotubes (CNTs) melt flow under varying shear and cooling conditions and found that shear significantly increased nucleation density, but rapid post-shear cooling resulted in incomplete lamellar growth [52]. Similarly, Ru et al. studied neat PLA under combined shear and pressure and reported that shear induced oriented nuclei and the development of β-form-dominant crystalline structures. However, high pressure during subsequent cooling suppressed their growth, producing imperfect crystalline regions [53]. These results highlight that the evolution of crystalline morphology in PLA-2OA is governed not solely by the presence of a nucleating agent but by the combined influence of cooling rate, pressure evolution, and flow-induced orientation, each acting on distinct timescales during solidification and contributing to the overall degree of crystallinity.

#### 3.2.3. Crystalline Morphology

**Figure 13:**
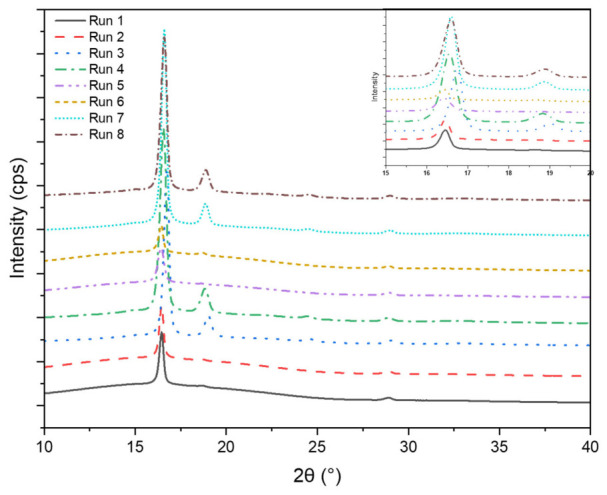
XRD patterns for PLA-2OA samples; close-up view of 2θ = 15–20 ° is also presented in an inserted figure.

The X-ray diffraction (XRD) patterns of the PLA–2OA samples, as presented in Figure 13, exhibit a dominant reflection near 2θ ≈ 16.4–16.6°, characteristic of the (200) planes of semicrystalline poly (L-lactic acid). Reference α and α′ crystal forms are typically reported at 16.62° and 16.44°, respectively [54,55]. The measured peak positions for all samples fall between these two values, indicating the coexistence of both polymorphs. The α′ modification corresponds to a less ordered orthorhombic lattice with greater interplanar spacing (d ≈ 5.39 Å), while the α phase possesses tighter chain packing (d ≈ 5.33 Å) and higher crystallographic order.

In this study, the mold was maintained at 95 °C, a temperature well above the glass transition but below the melting range of PLA. Under such warm-mold conditions, the polymer melt remains in a semi-solid, high-mobility state for a relatively long period before solidification, providing a favorable environment for in-mold crystallization. Despite this, a strong dependence on packing pressure was observed (c.f.: Table 13). Samples molded at low packing pressure (Runs 3, 4, 7, 8) exhibited markedly higher peak intensities (≈1.17–1.51 × 10^6^ cps) and crystallinity values (~52%), with peak positions near 2θ = 16.60–16.64°, indicative of an α-phase–dominant morphology. In contrast, high-pressure samples (Runs 1, 2, 5, 6) displayed weaker intensities (<3 × 10^5^ cps) and slightly shifted peaks near 16.41–16.45°, consistent with the predominance of the disordered α′ phase. The corresponding increase in d-spacing (5.34 → 5.39 Å) reflects reduced lattice regularity and incomplete crystal growth.

The α′ → α transformation in PLA is known to depend on both temperature and molecular mobility. Tsuji and Ikada demonstrated that α′ crystals form under rapid cooling or low crystallization temperatures (<120 °C), while annealing near 150–160 °C allows rearrangement into the stable α form [54]. Zhang et al. further showed that this transition involves a gradual reorganization of 10_3_ helices as chain mobility increases [55]. Although the present mold temperature (95 °C) is below the equilibrium transition window, it lies within the temperature range where α′ nuclei can reorganize toward α if molecular mobility and residence time permit. The low packing pressure appears to slow cooling and reduce molecular constraint, extending the period during which rearrangement is possible and enabling partial conversion to α. In contrast, high packing pressure imposes mechanical compression that restricts chain mobility. In such conditions, molecules are trapped in the α′ phase even in a warm cavity. Similar pressure-dependent polymorphism has been reported in other studies. Puiggali et al. observed that externally applied stress and rapid solidification in PLA films inhibit the α′ → α transition, leading to broader and weaker diffraction peaks [56]. Salač et al. investigated PLA containing orotic acid and talc nucleating agents and found that while such additives promote early α′ nucleation, the α-phase fraction is controlled by local cooling and pressure history more dominantly [24]. These findings conform to the observations in this study. The 95 °C mold provided sufficient thermal energy for crystal reorganization, but only the low-pressure condition allows the necessary chain mobility for the α phase to develop.

Taken together, the XRD results demonstrate that both the crystallinity and crystal form of PLA–2OA are strongly affected by packing pressure through its influence on cooling kinetics and molecular mobility. The low-pressure samples exhibit higher crystallinity, narrower diffraction peaks, and smaller d-spacing values indicative of better-ordered α lamellae, whereas high-pressure samples are dominated by the kinetically trapped α′ modification. These morphological outcomes are fully consistent with the DSC results and explain the observed differences in mechanical performance discussed in the following section.

#### 3.2.4. Tensile Properties

**Figure 14:**
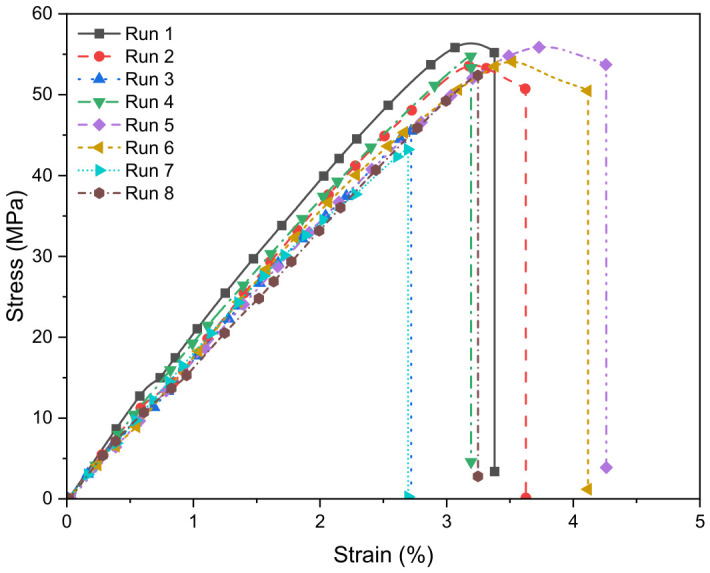
Stress vs. strain curves of PLA-2OA samples.

**Figure 15:**
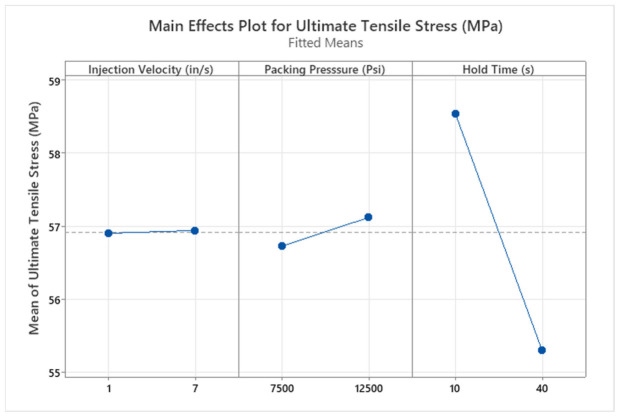
Main effects factorial plots for ultimate tensile stress for PLA-2OA samples.

**Figure 16:**
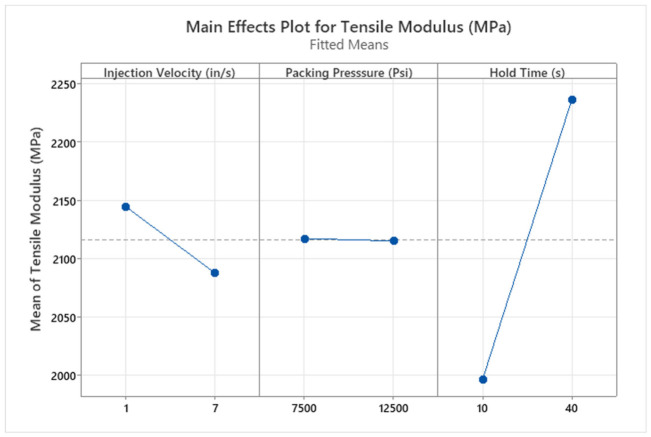
Main effects factorial plots for Young’s modulus of PLA-2OA samples.

The tensile properties of PLA-2OA samples are summarized in Table 14, and a stress vs. strain curve is presented in Figure 14. ANOVA results showed that hold time was the only statistically significant factor for the modulus of elasticity (*p* = 0.002), where longer hold time produced higher stiffness. The measured moduli ranged from approximately 2.00 to 2.30 GPa across all samples. In contrast, ultimate tensile stress (UTS) decreased significantly with increasing hold time (*p* = 0.025), with measured UTS values spanning ~51.7 to 59.6 MPa. Factorial plots of the DOE run are presented in Figure 15 and Figure 16. Yield strain also exhibited a slight downward trend at higher hold times, suggesting decreasing ductility.

DSC analysis indicated that crystallinity did not vary statistically significantly with hold time, so the mechanical trends are likely related to differences in consolidation and stress development during solidification rather than to changes in crystal content.

The increase in tensile modulus with longer hold time suggests that extended pressure during the early stages of solidification leads to more effective densification of the skin layer. A more compact surface region reduces void content and increases continuity in the load-bearing zone during tension. Similar stiffening effects driven by improved microstructural cohesion have been reported in biodegradable polymer systems. Jang et al. demonstrated that enhanced structural continuity in polymer networks increased the modulus even when crystallinity remained unchanged, highlighting the importance of densification and improved load-transfer pathways [57]. Although their material system differs from the small-molecule-modified PLA examined here, the mechanistic principle remains relevant: improved structural packing can elevate modulus without requiring changes in crystallinity. In contrast, the decrease in tensile strength with increasing hold time is consistent with the development of residual stresses and local brittleness in regions that solidify under prolonged pressure. Ghazvini et al. observed comparable behavior in PLA-based materials, where reduced relaxation during cooling resulted in increased rigidity but lower tensile strength due to early crack initiation [58]. Mazur et al. reported similar reductions in strength in PHBV-based systems with constrained microstructures, where limited molecular mobility during solidification promoted brittle failure despite adequate stiffness [59]. In the PLA–OA samples, a more consolidated skin layer formed during extended packing appears to retain higher internal stress, reducing the material’s ability to deform plastically and lowering tensile strength. The presence of OA may also contribute to this behavior by influencing structural development rather than the final crystalline fraction. As discussed in the review by Siddiqui et al. [60], small-molecule additives can encourage earlier structural organization and reduce relaxation time during cooling. In systems where the matrix solidifies before stresses dissipate, stiffness can increase while strength decreases due to increased brittleness. A similar effect may be present in the PLA–OA blend investigated here, where OA may slightly shift the solidification window and accentuate the differences between modulus and strength.

#### 3.2.5. Flexural Properties

**Figure 17:**
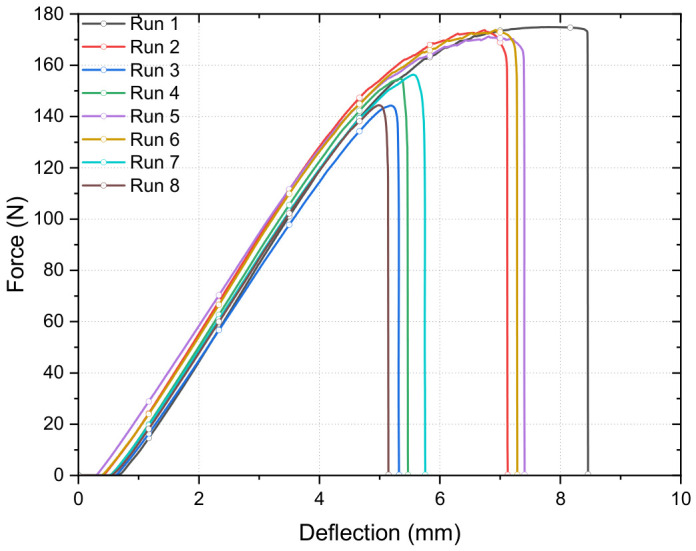
Compression force vs. deflection curves of PLA-2OA samples.

**Figure 18:**
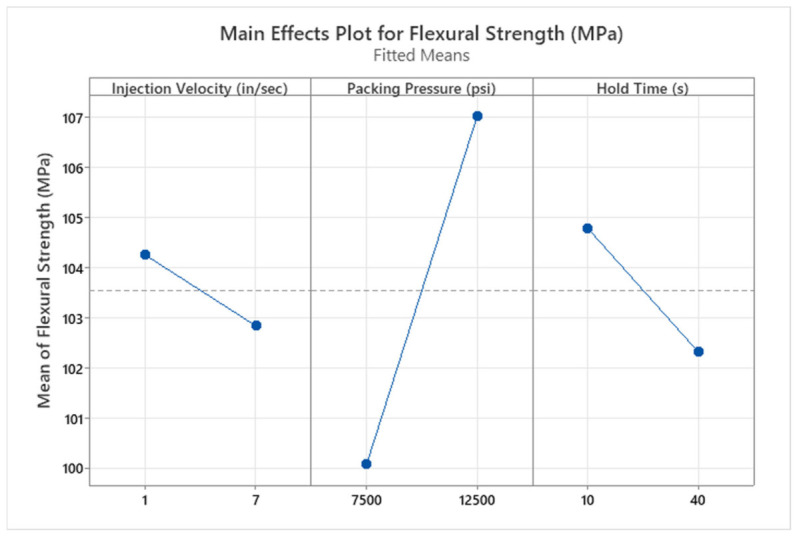
Main effects factorial plots for flexural strength of PLA-2OA samples.

**Figure 19:**
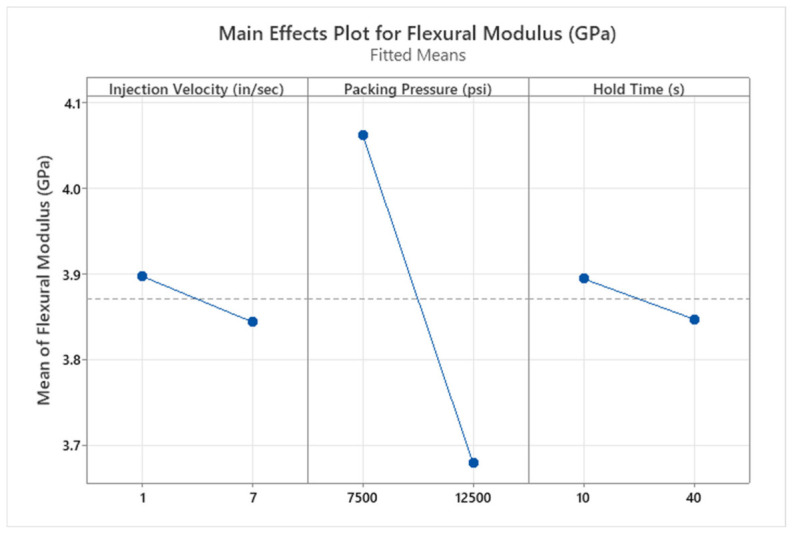
Main effects factorial plots for flexural modulus of PLA-2OA samples.

Flexural testing revealed distinct responses of flexural strength and flexural modulus to the molding parameters. Across all experiments, flexural modulus ranged from approximately 3.64 to 4.12 GPa, and flexural strength ranged from about 93.9 to 107.7 MPa, as presented in Table 15 and Figure 17. ANOVA identified packing pressure as the most significant factor for both properties, with hold time also significantly affecting flexural strength and injection velocity significantly influencing flexural modulus. Examination of the main effects showed that flexural modulus decreased as packing pressure, hold time, and injection velocity increased, while flexural strength increased with packing pressure but decreased with higher duration and velocity, as presented in Figure 18 and Figure 19.

The increase in flexural strength with packing pressure indicates that higher pressure improved consolidation in the outer layers, which has the most significant contribution to failure during bending. Enhanced compaction reduces voids and produces a more cohesive skin region. Ghazvini et al. reported similar improvements in strength for PLA/PBS blends, where increased cohesion in the surface region enhanced resistance to crack initiation [58]. A similar mechanism appears to operate in the present PLA–OA system, where improved surface integrity at higher packing pressure contributes to increased flexural strength.

In contrast, flexural modulus decreased as packing pressure increased. DSC results revealed that crystallinity decreased substantially with increasing pressure, falling from approximately 52% at low pressure to roughly 29% at high pressure. This reduction in crystalline content likely plays a key role in the decrease in flexural modulus. A significant loss in crystallinity reduces the rigidity of the material through the thickness. Mazur et al. described a related trend in PHBV composites where decreases in crystallinity corresponded to lower flexural modulus [59]. In the PLA–OA samples, the large reduction in crystallinity provides a direct explanation for the drop in modulus with increasing packing pressure.

The influence of hold time on flexural strength and modulus can be attributed to stress development rather than crystalline effects, since the degree of crystallinity was not sensitive to duration. Increased duration reduced flexural strength, suggesting that longer pressure application limited relaxation and promoted the formation of stress gradients in the surface and subsurface layers. These gradients may reduce fracture resistance, consistent with observations reported by Ghazvini et al., where limited relaxation produced more brittle failure [58]. The decrease in flexural modulus with increasing duration also suggests that residual stresses disrupt coordinated bending deformation.

Lastly, injection velocity further contributed to reductions in flexural modulus and showed a decreasing trend in flexural strength. Higher velocity increases shear during filling and promotes rapid solidification near the surface. Siddiqui et al. noted that rapid structural development in bioplastics can stiffen local domains while reducing overall mechanical uniformity, which may reduce modulus and promote early failure [60].

#### 3.2.6. Heat Deflection Temperature

The PLA–OA specimens exhibited a wide range of heat deflection temperatures, with measured HDT values spanning from approximately 58 to 131 °C, as presented in Table 16. This represents a substantial improvement over neat PLA, which typically exhibits HDT values near 55–60 °C under similar loading conditions. Several runs in the present study exceed this baseline by a large margin, demonstrating that the combination of orotic acid and specific molding conditions can produce meaningful enhancements in thermal performance.

A comparison of the degree of crystallinity and HDT data in Table 16 shows an overall positive relationship between crystal development and thermal performance. Samples exhibiting crystallinity values near 20–30% generally displayed HDT values below 75 °C, whereas samples reaching approximately 48–50% crystallinity demonstrated the potential for substantially higher thermal resistance. This trend is consistent with the established role of crystalline regions in restricting chain mobility above the glass transition temperature and improving resistance to thermally induced deformation. However, the similar crystallinity values observed for Runs 3, 4, 7, and 8 indicate that crystallinity alone cannot fully explain the HDT response, suggesting that additional structural and processing-related factors contribute to thermal stability.

Runs 3 and 4 exhibited very high HDT values of approximately 100 °C and 131 °C, respectively. These samples were molded under low packing pressure and long hold time, a combination that produced the highest crystallinity measured by DSC and also promoted extensive α-phase formation according to the XRD analysis. The α crystalline form of PLA is known to be more thermally stable and structurally ordered than the α′ form, and the combination of high crystallinity and strong α-phase development provides a favorable foundation for higher HDT. The long hold time in Runs 3 and 4 also provided additional time for lamellae to thicken and organize, which further contributes to thermal stability and delays the onset of deflection under load.

Although Runs 7 and 8 also displayed relatively high crystallinity and a strong presence of α-phase, their HDT values did not approach those of Runs 3 and 4. This indicates that α-phase content and crystallinity, while important, do not fully determine the HDT response. One likely explanation is that the shorter hold times in Runs 7 and 8 limited the extent of lamellar thickening and crystal perfection, even though α-phase was present. XRD confirms the crystal form, but not the crystal quality, lamellar thickness, or degree of perfection, all of which influence thermal resistance. Long packing times under low pressure can support the development of thicker, more stable lamellae, whereas shorter durations tend to produce crystallites that are abundant but less thermally robust.

Another factor influencing the differences between these sample sets is residual stress. Samples molded under shorter hold time or more rapid cooling, such as Runs 7 and 8, are more likely to retain higher internal stresses in the skin and subsurface regions. These stresses can lower the effective HDT by promoting earlier softening or deformation under load. Similar effects have been reported in biodegradable polymers, where insufficient relaxation during solidification reduced thermal and mechanical stability even when crystallinity and crystal form were adequate, as noted by Ghazvini et al. and Mazur et al. [58,59].

## 4. Conclusions

This study investigated the influence of orotic acid nucleation and injection molding conditions on the crystallization behavior and thermo-mechanical performance of PLA. By integrating DSC, XRD, tensile, flexural, and HDT measurements, the results revealed how packing pressure, hold time, and injection velocity shape the development of crystalline structure, crystal form, lamellar organization, and residual stress in molded PLA–OA parts.

Incorporating 2 wt% orotic acid significantly enhanced PLA crystallization, increasing crystallinity from approximately 17–23 percent in neat PLA to as high as 52–53 percent under low-pressure, long-duration molding conditions. Mechanical performance was strongly influenced by packing conditions: tensile modulus increased with longer hold time, while flexural strength increased with packing pressure. Flexural modulus and tensile strength decreased under conditions that promoted structural heterogeneity or residual stress, indicating that mechanical behavior is governed by both crystal development and the stress state established during solidification. Heat deflection temperature improved substantially in the PLA–OA system, reaching 100–131 °C in optimized samples, which is 40–70 °C higher than the heat deflection temperature of neat PLA samples.

This study demonstrates that orotic acid is an effective bio-based nucleating agent that enables high-crystallinity, high-HDT PLA through standard injection molding without the need for post-annealing. By optimizing packing pressure and duration, PLA–OA can achieve thermal performance suitable for microwave reheating, hot-filling, and other moderate-temperature applications currently dominated by petroleum-based plastics. These results highlight a practical route to expanding the utility of PLA through formulation–processing control and underscore the potential of nucleated PLA systems for durable, thermally stable, and more sustainable products.

## Figures and Tables

**Table 1:** Key physical and thermal properties of PLA 3100HP.

Properties	Units	Values
Specific Gravity		1.24
Melt Flow Index (210 °C, 2.16 kg)	g/10 min	24
Relative Viscosity (in 1 g/dL chloroform, 30 °C)		3.1
Melting Temperature	°C	165–180
Glass Transition Temperature	°C	62
Crystallization Temperature	°C	86–124

**Table 2:** Injection molding conditions for neat PLA and PLA-2OA specimens.

Material	Set Point	V: Injection Velocity (in/s)	P: Hold Pressure (psi)	T: Hold Time (s)
Neat PLA	+	7	10,000	12
	Mid	4	7500	9
	-	1	5000	6
PLA-2OA	+	7	12,500	40
	Mid	4	10,000	20
	-	1	7500	10

**Table 3:** Design of experiment (DOE) parameters and levels.

Run Order	V	P	T
1	+	+	+
2	-	+	+
3	+	-	+
4	-	-	+
5	+	+	-
6	-	+	-
7	+	-	-
8	-	-	-

**Table 4:** Degradation temperatures for neat PLA samples.

Run Order	Degradation Onset Temperature (°C)	90% Weight Loss Temperature (°C)
1	333.33	345.21
2	333.28	344.76
3	332.12	340.21
4	327.49	346.13
5	325.57	346.60
6	324.86	344.26
7	330.75	351.49
8	332.76	346.08

**Table 5:** Melting enthalpy and cold crystallization enthalpy of neat PLA samples.

Run Order	Cold Crystallization				Melt				Enthalpy Difference, ΔH_m_ − ΔH_cc_ (J/g)	
	Enthalpy, ΔH_cc_ (J/g)		Temperature, T_cc_ (°C)		Enthalpy, ΔH_m_ (J/g)		Temperature, T_m_ (°C)			
	Average	Std. Dev	Average	Std. Dev	Average	Std. Dev	Average	Std. Dev	Average	Std. Dev
1	22.11	3.53	102.29	1.81	41.62	2.67	178.51	0.77	19.51	3.06
2	21.65	0.34	100.41	0.86	43.21	0.75	175.31	4.64	21.56	0.82
3	23.71	2.45	101.23	1.01	40.46	4.66	178.97	2.34	16.75	4.10
4	23.22	3.05	101.74	1.36	41.92	2.56	177.92	0.96	18.70	5.56
5	23.71	1.24	100.46	0.19	43.04	1.10	177.52	0.22	19.33	1.99
6	23.47	0.81	101.79	1.11	39.6	1.57	179.81	1.76	16.13	2.20
7	23.39	1.35	96.77	1.07	43.93	1.69	177.62	0.48	20.54	0.62
8	23.72	1.43	101.28	1.06	40.33	2.93	179.23	0.69	16.61	4.36

**Table 6:** Degree of crystallinity of neat PLA samples.

Run Order	Degree of Crystallinity (%)	
	Average	Std. Dev
1	20.97	3.29
2	23.19	0.88
3	18.02	4.41
4	20.10	5.98
5	20.78	2.14
6	17.34	2.37
7	22.09	0.67
8	17.86	4.69

**Table 7:** Tensile properties of neat PLA samples.

Run Order	Ultimate Tensile Stress (MPa)		Yield Strain (%)		Modulus of Elasticity (GPa)	
	Average	Std. Dev	Average	Std. Dev	Average	Std. Dev
1	62.46	2.88	3.93	0.11	1.99	0.11
2	59.34	1.17	3.76	0.17	2.13	0.05
3	57.92	1.79	3.74	0.1	2.09	0.1
4	56.43	0.94	3.71	0.09	2.54	0.13
5	67.57	3.05	3.7	0.12	2.41	0.04
6	62.97	2.84	3.61	0.06	1.85	0.18
7	57.47	1.12	3.66	0.14	2.06	0.08
8	59.75	2.08	3.67	0.2	2.03	0.07

**Table 8:** Flexural properties for neat PLA samples.

Run Order	Flexural Modulus (GPa)		Flexural Strength (MPa)	
	Average	Std. Dev	Average	Std. Dev
1	3.30	0.11	112.36	2.51
2	3.36	0.06	113.67	1.03
3	3.18	0.03	92.24	4.84
4	3.24	0.12	101.57	1.82
5	3.40	0.06	114.79	1.12
6	3.40	0.07	115.69	1.13
7	3.16	0.07	102.08	2.52
8	3.16	0.08	98.45	1.75

**Table 9:** Heat deflection temperature for neat PLA samples.

Run #	Degree of Crystallinity (%)	Heat Deflection Temperature (°C)	Final Deflection (mm)
1	19.51	58.14	10.52
2	21.56	58.54	10.50
3	16.75	58.08	10.50
4	18.70	58.22	10.50
5	19.33	58.02	10.52
6	16.13	58.57	10.51
7	20.54	57.23	10.49
8	16.61	58.73	10.51

**Table 10:** Degradation temperature of PLA-2OA samples.

Run	Degradation Onset Temperature (°C)	90% Weight Loss Temperature (°C)
1	329.01	343.04
2	327.92	341.28
3	328.71	342.08
4	330.4	344.61
5	329.42	343.08
6	328.71	341.90
7	330.06	346.44
8	329.64	340.57

**Table 11:** Melting enthalpy and cold crystallization enthalpy of PLA-2OA samples.

Run Order	Cold Crystallization				Melt				Enthalpy Difference, ΔH_m_ − ΔH_cc_ (J/g)	
	Enthalpy, ΔH_cc_ (J/g)		Temperature, T_cc_ (°C)		Enthalpy, ΔH_m_ (J/g)		Temperature, T_m_ (°C)			
	Average	Std. Dev	Average	Std. Dev	Average	Std. Dev	Average	Std. Dev	Average	Std. Dev
1	22.82	1.92	91.93	1.01	49.70	1.90	175.51	0.48	27.51	0.27
2	22.73	1.61	91.39	0.56	46.45	2.41	176.13	0.25	22.71	4.02
3	1.23	0.18	-	-	49.54	0.42	176.02	0.38	48.31	0.27
4	1.02	0.24	-	-	50.17	1.52	175.71	0.44	49.16	0.90
5	25.74	1.68	90.60	0.71	47.20	1.03	176.53	0.23	21.55	1.29
6	24.23	0.70	90.93	0.62	47.75	1.28	177.83	0.04	23.52	1.98
7	2.27	1.09	-	-	50.60	0.37	175.77	0.35	48.34	1.03
8	0.96	0.25	-	-	50.53	0.70	175.11	0.30	49.58	0.94

**Table 12:** Degree of crystallinity for PLA-2OA samples.

Run Order	Degree of Crystallinity (%)	
	Average	Std. Dev
1	29.58	0.29
2	24.32	4.32
3	51.95	0.29
4	52.86	0.97
5	23.17	1.39
6	25.29	2.13
7	51.97	1.10
8	53.31	1.01

**Table 13:** Summary of XRD peak parameters and degree of crystallinity of PLA-2OA samples.

	2θ (°)	Std. Dev (°)	d (Å)	Peak Intensity (cps)	Degree of Crystallinity (%)
α	16.62		5.33		
α′	16.44		5.39		
Run 1	16.45	0.01	5.39	413,826	29.58
Run 2	16.53	0.01	5.36	126,790	24.32
Run 3	16.64	0.01	5.29	1,296,317	51.95
Run 4	16.56	0	5.35	1,426,386	52.86
Run 5	16.41	0.01	5.40	267,778	23.17
Run 6	16.42	0.01	5.39	215,424	25.29
Run 7	16.60	0	5.34	1,508,739	51.97
Run 8	16.60	0	5.34	1,172,954	53.31

**Table 14:** Tensile properties of PLA-2OA samples.

Run Order	Ultimate Tensile Stress (MPa)		Yield Strain (%)		Modulus of Elasticity (GPa)	
	Average	Std. Dev	Average	Std. Dev	Average	Std. Dev
1	55.53	1.85	3.25	0.06	2.21	0.14
2	57.01	2.39	3.29	0.17	2.20	0.06
3	51.75	4.65	2.89	0.27	2.21	0.79
4	56.43	3.78	3.00	0.28	2.30	0.13
5	59.22	2.23	3.63	0.20	2.09	1.54
6	56.15	3.40	3.57	0.11	2.05	1.34
7	59.64	5.30	3.25	0.50	2.24	0.09
8	57.65	1.24	3.07	0.18	2.00	0.08

**Table 15:** Flexural properties of PLA-2OA samples.

Run	Flexural Modulus (GPa)		Flexural Strength (MPa)	
	Average	Std. Dev	Average	Std. Dev
1	3.64	0.08	105.67	1.51
2	3.69	0.04	107.47	0.88
3	3.95	0.10	93.88	5.26
4	4.12	0.10	102.23	1.99
5	3.68	0.07	107.29	1.46
6	3.71	0.09	107.65	1.72
7	4.11	0.03	104.51	1.64
8	4.08	0.04	99.68	2.00

**Table 16:** Heat deflection properties of PLA-2OA samples.

Run	Degree of Crystallinity (%)	Heat Deflection Temperature (°C)	Final Deflection (mm)
1	27.51	59.23	10.52
2	22.71	58.47	10.51
3	48.31	100.13	10.50
4	49.16	131.40	10.54
5	21.55	58.63	10.52
6	23.52	71.09	10.52
7	48.34	69.35	10.42
8	49.58	65.44	10.50

## Data Availability

The raw data supporting the conclusions of this article are contained within the article. Further inquiries will be made available by the authors on request.
